# LRRK2 Biology from structure to dysfunction: research progresses, but the themes remain the same

**DOI:** 10.1186/s13024-019-0344-2

**Published:** 2019-12-21

**Authors:** Daniel C. Berwick, George R. Heaton, Sonia Azeggagh, Kirsten Harvey

**Affiliations:** 1 School of Health, Life and Chemical Sciences, The Open University, Walton Hall, Milton Keynes, MK7 6AA UK; 2 Department of Pharmacology, UCL School of Pharmacy, University College London, 29-39 Brunswick Square, London, WC1N 1AX UK

**Keywords:** LRRK2, Parkinson’s disease, Rab29, autophagy, lysosomes, endocytosis, Golgi, Wnt, microtubules

## Abstract

Since the discovery of leucine-rich repeat kinase 2 (LRRK2) as a protein that is likely central to the aetiology of Parkinson’s disease, a considerable amount of work has gone into uncovering its basic cellular function. This effort has led to the implication of LRRK2 in a bewildering range of cell biological processes and pathways, and probable roles in a number of seemingly unrelated medical conditions. In this review we summarise current knowledge of the basic biochemistry and cellular function of LRRK2. Topics covered include the identification of phosphorylation substrates of LRRK2 kinase activity, in particular Rab proteins, and advances in understanding the activation of LRRK2 kinase activity via dimerisation and association with membranes, especially via interaction with Rab29. We also discuss biochemical studies that shed light on the complex LRRK2 GTPase activity, evidence of roles for LRRK2 in a range of cell signalling pathways that are likely cell type specific, and studies linking LRRK2 to the cell biology of organelles. The latter includes the involvement of LRRK2 in autophagy, endocytosis, and processes at the *trans*-Golgi network, the endoplasmic reticulum and also key microtubule-based cellular structures. We further propose a mechanism linking LRRK2 dimerisation, GTPase function and membrane recruitment with LRRK2 kinase activation by Rab29. Together these data paint a picture of a research field that in many ways is moving forward with great momentum, but in other ways has not changed fundamentally. Many key advances have been made, but very often they seem to lead back to the same places.

## Background

Leucine-rich repeat kinase 2 (LRRK2) is an enigmatic protein that has been at the centre of an increasing amount of research since its discovery in 2004. Although LRRK2 has been implicated in a number of human diseases, the basic function of this protein remains poorly understood. Debates span all levels of research; from biochemistry – how do the two enzymatic activities of LRRK2 relate to each other, and what effects do disease-causing mutation have? – to cell biology – what processes does LRRK2 mediate, and what are its phosphorylation substrates? Even the cell types this protein is most relevant to are under discussion. The study of LRRK2 continues to produce more questions than answers.

In this review we summarise the current state of the LRRK2 field, covering first the connections between LRRK2 and a surprising number of clinical conditions, before progressing to its mode of action and the cell biological processes it mediates. Although many details are missing and the field remains a long way from agreement, this is an exciting time for LRRK2 biology. Important advances have been made in distinct areas providing some consensus and a feeling that the field has momentum. In particular, breakthroughs relevant to disease treatment may be close.

### LRRK2 in disease

In this section we summarise the genetic connections between the *LRRK2* gene and human disease, starting first with the condition that LRRK2 is most strongly linked to: Parkinson’s disease (PD).

PD is the second most common neurodegenerative disease worldwide, with a lifetime risk estimated to be around 2% [1, 2]. Initially described by James Parkinson in 1817 as a “shaking palsy” [3], PD remains incurable 200 years later. The major risk factor is age, and since the world’s population is ageing, understanding the underlying PD pathomechanism is increasingly important.

Over the last 15-20 years a considerable amount of work has gone into determining the genetic causes of PD. Although PD is usually sporadic or idiopathic, it has long been known that around 1 in 10 PD patients have a family history of PD, so research initially focused on families who carry gene mutations that are sufficient to cause PD (i.e. monogenic forms of PD). More recently, PD genetics has expanded to more powerful genome-wide association studies (GWAS) that compare genetic markers from thousands of individuals with sporadic PD with genetic markers from similarly large numbers of healthy controls. GWAS are able to identify significant differences in frequency of particular SNPs that nominate loci containing gene variants associated with PD incidence. Some identified risk variants may be insufficient to cause disease by themselves, but can still have a significant impact on an individual’s lifetime risk of developing the condition.

In 2004 research into familial PD led two groups working independently to clone the gene that became known as *LRRK2* [4, 5]. Subsequent work has identified at least 9 missense mutations in *LRRK2* that appear sufficient to cause PD (i.e. pathogenic mutations), as well as other missense changes that affect PD risk, including both pathogenic and protective risk variants. We expand upon these below. Pathogenic *LRRK2* variants have been suggested to represent the largest known cause of PD worldwide, although this is hard to know for sure given that incidence varies between populations and not all populations have been thoroughly surveyed. The highest incidence is in parts of North Africa, where *LRRK2* mutations cause as much as 40% of all PD cases [6].

More recently *LRRK2* has been linked to PD a second time through GWAS [6]. These studies have repeatedly shown linkage of PD risk to *LRRK2*, and meta-analysis indicates *LRRK2* is one of the more important genomic loci influencing the condition [7]. Thus, *LRRK2* mutations make a large contribution towards both sporadic and familial forms of PD.

Remarkably, *LRRK2* has also been connected genetically to a number of chronic inflammatory conditions, beginning in 2008 with linkage to Crohn’s disease (CD), an inflammation of the terminal ileum, which was found via meta-analysis with subsequent replication of three separate GWAS investigations [8]. Initial studies were unable to distinguish between *LRRK2* and the neighbouring *MUC19* gene, which arguably delayed interest in *LRRK2* in this context. Although the linkage is relatively weak compared to other CD genes, the observation has been reproduced in a number of studies, e.g. [9–11], and very persuasively, both pathogenic and protective *LRRK2* variants have been reported [9]. It is worth noting that CD is one of two distinct chronic inflammatory intestinal disorders that are grouped together as inflammatory bowel disease, the other being ulcerative colitis, an inflammation of the colon [12]. This has led to *LRRK2* sometimes being reported as a risk factor for inflammatory bowel disease, even though linkage is stronger (and may be specific) to CD.

Just a year later, linkage to *LRRK2* was reported in GWAS of Chinese leprosy patients [13], a result that has been replicated in some [14, 15] but not all subsequent studies [16]. Leprosy (also known as Hansen’s disease) is a chronic inflammatory condition caused by *Mycobacterium leprae* infection of the skin and peripheral nerves. Fascinatingly, this work also implicated a number of genes in leprosy risk that had previously been linked to CD, including *NOD2* and *RIPK2* [13, 17], suggesting that these seemingly unrelated conditions may have similar pathomechanisms [17]. By contrast, similarities between leprosy and tuberculosis (TB), the third inflammatory disease linked to *LRRK2*, were already well established when this connection was reported via a meta-analysis of nine separate GWAS investigations of TB patients [18]. Unlike leprosy, TB affects the lungs, but both conditions are caused by mycobacterial infection and similarities between their genetic risks have been known since the late 1990’s [19, 20]. The lack of any replication studies and the relatively weak linkage that is implied by meta-analysis of nine studies means the role of *LRRK2* in TB should be treated with caution. Nonetheless, the similarities of TB to leprosy, and a recent publication demonstrating elegantly that LRRK2 kinase activity affects *Mycobacterium tuberculosis* infection *in vitro* and in mouse models make this a very interesting story to follow [21].

Taken together with the involvement of LRRK2 in a number of immune cell-relevant signalling pathways, which we expand upon below, the GWAS implication of *LRRK2* in the pathogenesis of three separate chronic inflammatory conditions creates a powerful body of work arguing for an essential function of LRRK2 in inflammatory responses that have potential implications for PD. Indeed, the connection between LRRK2 and CD forms part of a body of evidence that has been used to create a theory that PD may be a low-grade inflammatory bowel disease [22]. We would not dispute the strength of this argument, but it does not reconcile the linkage of *LRRK2* to leprosy and TB, which are in general not primarily gut disorders. We also note a report of elevated LRRK2 expression in the nasal linings of individuals with chronic rhinosinusitis [23]. It may thus be the case that it is chronic inflammation more generally and not specifically in the gut that increases the risk of PD. Alternatively, the roles of LRRK2 in these inflammatory conditions and in PD may yet be unrelated.

A final category of disease linked to *LRRK2* is cancer. Individuals with the most common pathogenic *LRRK2* mutation, G2019S, have been reported to have an increased risk of developing cancers [24–26] although this is disputed [27, 28]. This link to cancer is slightly surprising, as it is fairly well established that individuals with PD have a reduced incidence of almost all cancers apart from melanoma and other skin cancers [29]. As such the role of LRRK2 in cancer remains controversial, but serves to further the idea that LRRK2 is involved in more processes than are suggested by its links to PD alone.

### LRRK2 protein structure and function

LRRK2 is a highly unusual protein, containing four protein-protein interaction domains, as well as domains conferring two distinct enzymatic activities [6]. As defined by its kinase domain, LRRK2 is a serine-threonine kinase capable of autophosphorylating residues elsewhere in LRRK2, as well as phosphorylating a select group of heterologous substrates (see next section). The second enzymatic activity is GTPase activity, which is mediated by the Roc (Ras of complex proteins) domain. Throughout evolution Roc domains are always accompanied by a COR (C-terminal of Roc) domain [30] and thus, even though both Roc and COR form distinct globular structures and are individual domains in the conventional sense, they are functionally inseparable and considered by many a RocCOR tandem domain. The mode of action of the LRRK2 GTPase is discussed in the next section.

**Fig. 1:**
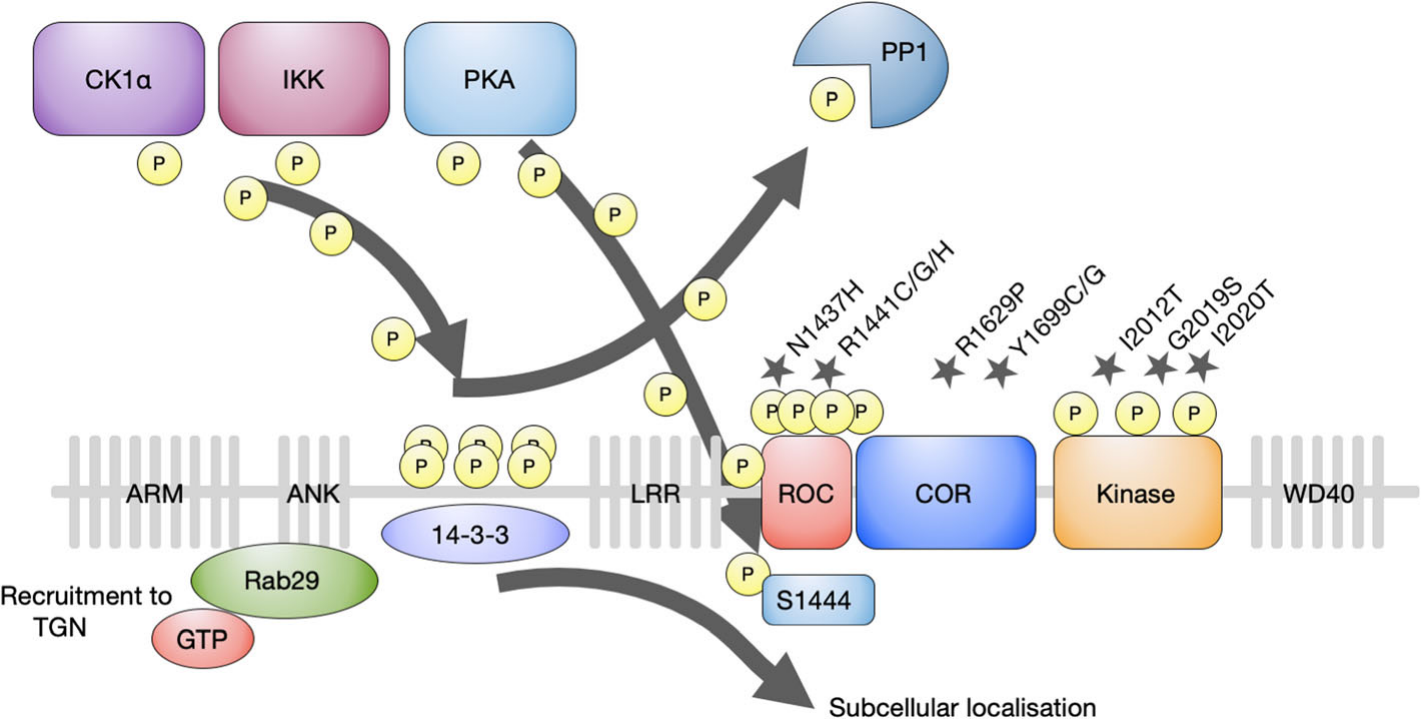
LRRK2 domain structure and function. LRRK2 contains a catalytic core, conferring GTPase activity via the RocCOR domain and kinase activity, embedded in ARM, ANK, LRR and WD40 protein-protein interaction domains. LRRK2 mutations are indicated with a star above the domain structure. LRRK2 is regulated by autophosphorylation of the kinase and Roc domain. Heterophosphorylation by CK1α, IKK and PKA, and dephosphorylation by PP1 regulates interaction with 14-3-3 proteins affecting LRRK2 localisation. Rab29 recruits LRRK2 to the TGN further depicted in detail in Fig. 2.

The structure of LRRK2 is depicted in Fig. 1. The key points, which we expand upon in the next two sections, are the two enzymatic activities and how they relate to each other, the capacity of LRRK2 to switch between dimeric and monomeric forms and how this may affect its function, as well as the remarkable number of reported interacting proteins that suggest LRRK2 likely acts in larger multiprotein complexes.

### LRRK2 kinase activity

Of the two LRRK2 enzymatic activities, the majority of work has focussed on its kinase activity. There are a number of reasons for this, the most important being the early observation that the most common pathogenic variant, G2019S, displays a modest but reproducible elevation of kinase activity, when assayed by measuring the phosphorylation of a substrate peptide *in vitro* [32]. This led to the hypothesis that all pathogenic mutations would be gain-of-function that cause PD via hyperphosphorylation of substrate proteins, which in turn triggered considerable efforts to develop pharmacological inhibitors of LRRK2 kinase activity.

The path from these initial kinase assays to the position we are in today has been far from plain sailing. Major problems included the failure of other pathogenic mutations to display convincingly increased kinase activity *in vitro*, and the remarkable difficulties in identifying agreed physiological substrates. For a long time, increased kinase activity was the dominant idea in the LRRK2 field, but with a growing dose of scepticism: perhaps the elevated kinase activity of G2019S *in vitro* was a red herring?

However the eventual identification of physiological substrates, first with the description of a robust LRRK2 autophosphorylation site, serine-1292 [33], and then, more significantly, with the description of a group of Rab small GTPases (Rab3A/B/C/D, Rab8A/B, Rab10, Rab12, Rab29, Rab35, Rab43) as heterologous substrates [34], has cleared up most of the doubt. Most notably, autophosphorylation of serine-1292 and Rab protein phosphorylation are both robustly and reproducibly increased by pathogenic LRRK2 variants, both *in vitro* and in cultured cells. Presumably, G2019S elevates LRRK2 kinase activity and thus increases the phosphorylation of physiological targets and small peptides alike, whereas the other pathogenic variants act independently of kinase activity, perhaps by facilitating interactions with substrate proteins. Phosphorylation of these Rab proteins by LRRK2 perturbs their ability to bind both upstream regulatory proteins and downstream effector proteins [34, 35], and convincing effects of Rab phosphorylation on cell biological processes, including ciliogenesis and rates of endocytosis, have been described [35, 36]. Full mechanistic details remain to be established, but it has been postulated that phosphorylated Rab proteins become trapped on intracellular membranes, unable to relocate to the compartments in which they are needed ]^37^]. We expand upon the consequences of Rab phosphorylation later in this review. In any case, after a fairly bumpy road, the increased kinase activity hypothesis looks to be correct, and a large amount of work is now focussed on LRRK2 and Rab protein phosphorylation, and enthusiasm for identifying new substrate proteins is renewed. It must also be said that the success of the Rab phosphorylation story appears to be justifying the considerable investment into developing LRRK2 kinase inhibitors as potential treatments for PD. LRRK2 kinase inhibitors have been reviewed by others (e.g. [38]) and are in early phase clinical trials; results are awaited with cautious optimism.

So, if Rabs are downstream of LRRK2 kinase activity, what is upstream? As we and others have argued, a major theme for LRRK2 appears to be as signalling scaffold [39, 40], and a number of signalling mechanisms have been reported to function up and downstream of LRRK2. But if we limit ourselves to pathways that specifically act on LRRK2 kinase activity we find ourselves returning to Rab GTPases and specifically Rab29. Rab29 (then known as Rab7L1) first entered the radar of LRRK2 researchers as a modifier of *LRRK2* PD risk, and a PD risk gene in its own right, that physically associates with LRRK2 in cells and rescues certain cellular phenotypes associated with the LRRK2 G2019S variant, which we expand upon below [41]. This interaction was confirmed shortly afterwards in an unbiased screen for novel LRRK2 binding proteins [42]. These and subsequent studies have shown that Rab29 recruits LRRK2 to the Golgi complex and this interaction appears to increase the kinase activity of LRRK2, as determined by both LRRK2 serine-1292 phosphorylation [33], and the phosphorylation of Rab substrate proteins [43, 44]. Interestingly, the phosphorylation of Rab29 by LRRK2 appears to weaken its ability to enhance LRRK2 kinase activity, suggesting that in addition to a Rab29-LRRK2-Rab signalling axis, there may also be a Rab29-LRRK2-Rab29 negative feedback mechanism [43].

Since these are recent developments, the consequences of LRRK2 activation by Rab29 are not yet fully understood, although we discuss its potential role in cell biological processes later in this article. Despite this, the potential impact on the study of LRRK2 of having Rab29 as a LRRK2 kinase activator makes this a very useful breakthrough indeed.

### LRRK2 GTPase activity

LRRK2 GTPase activity has received less attention than that of the kinase domain, yet the number of pathogenic mutations located in the Roc and COR domains indicate LRRK2 GTPase activity is no less important. As we have reviewed elsewhere [45], all pathogenic RocCOR mutations tested either increase affinity for GTP or decrease rates of GTP hydrolysis (or do both), all of which can be expected to lead to more LRRK2 in the GTP-bound state (as opposed to GDP-bound or not bound to guanine nucleotides). Corroborating this further, the R1398H Roc domain variant, which is protective against PD and CD [9], displays weakened GTP binding and an increased rate of GTP hydrolysis [9, 46].

However, the mechanism by which LRRK2 hydrolyses GTP to GDP and then recycles back to GTP-bound is still unclear, with many studies limited by just using the isolated Roc or RocCOR domains. Nonetheless, since there is a growing agreement that the COR domain is required for normal LRRK2 GTPase function, the consensus is that the Roc domain does not act in a manner analogous to small GTPases. This view is supported further by the lack of classical GAPs and GEFs for LRRK2. Although some enzyme kinetics studies suggest a requirement for additional proteins to facilitate GTP hydrolysis or guanine nucleotide exchange [47, 48], none of the GAPs and GEFs that have been proposed (ARHGEF7, ArfGAP1, and RGS2 [49–51]), are reported to bind directly to the Roc domain as would be expected for the GAPs and GEFs of a small GTPase. Instead, most theories for LRRK2 GTPase function are based around a so-called GTPase Activated by Dimerisation (GAD) model, which is drawn largely from studies using homologous RocCOR domain containing proteins. Inferences from experiments that use proteins from distantly related species must clearly be made with caution, and it is worth noting that the *C.tepidum* RocCOR protein in which most work has been done requires the intermolecular exchange of Roc domain lysine residues that are not present in human LRRK2 to form an active site [52]. Nonetheless the structure of the *C.tepidum* RocCOR fits very well to the structure of full-length human LRRK2 dimers as revealed by negative-stain electron microscopy [53], suggesting that at the very least, LRRK2 is a “GAD-like” GTPase.

In the GAD model, LRRK2 functions as a homodimer, with dimerisation mediated by the COR domain, creating a structure where the Roc domains of each LRRK2 molecule face each other [54]. It was previously assumed that GTP hydrolysis was achieved by the two Roc domains coming together, but more recent evidence contradicts this idea. Specifically, Deyaert and colleagues have shown that the isolated RocCOR domain is primarily dimeric when bound to GDP or when no nucleotides are present, but *monomeric* when GTP bound [31]. Based on this, they propose a model where LRRK2 is required to be in the GDP-bound state to dimerise, and the exchange of GDP for GTP triggers the dissociation of the dimer, with GTP hydrolysis occurring subsequently when monomeric [31, 54]. These observations must be treated with caution as they were not performed using full-length LRRK2, which has additional domains that may support dimerisation (in particular the C-terminal WD40 domain [55]), but they are fascinating. We discuss their implications for LRRK2 as a whole further in the next section.

As a final point of comment on LRRK2 GTPase activity, it is worth observing that although many proteins have been reported to bind the Roc domain, no heterologous interacting proteins that bind to the Roc domain when only in GTP- or GDP-bound states have been reported. This is perhaps slightly surprising. Guanine nucleotide-specific interactions with effector molecules are how small GTPases typically act, so it would not be unreasonable to hypothesise that one or more proteins functioning downstream of LRRK2 might interact with the Roc or RocCOR domains in a GTP- or GDP-dependent manner. Since it took such a long time for the LRRK2 field to agree on any widely accepted substrates of LRRK2 kinase activity, it would be unwise to exclude the possibility that GTPase effector proteins exist, but at present the most likely purpose of this enzymatic activity appears to be control of LRRK2 itself.

### Integrating LRRK2 kinase activity, GTPase activity and dimerisation – a single mechanism?

The proposed GTP/GDP-dependent switch between monomeric and dimeric forms of LRRK2 is intriguing. LRRK2 has long been known to exist in cells as both monomers and dimers, with evidence that the two LRRK2 species have different properties and subcellular locations. Specifically, dimeric forms of LRRK2 are enriched on intracellular membranes and possess enhanced kinase activity (as judged by *in vitro* assays of autophosphorylation), whereas monomeric LRRK2 is predominantly cytosolic with lower kinase activity [56–59]. LRRK2 dimerisation is clearly an important regulatory mechanism.

Nonetheless, these results also present a conflict. On the one hand, all pathogenic LRRK2 mutants display increased phosphorylation of Rab proteins, a phosphorylation event that takes place on intracellular membranes, which suggests pathogenic mutants are more likely to be dimeric. But on the other hand, pathogenic RocCOR mutants shift LRRK2 into a GTP-bound state, which based on Deyaert et al’s work predicts a preference for the monomeric form. Indeed, pathogenic mutations have been reported to weaken dimerisation of isolated RocCOR domain fragments [46, 60, 61], although not the full-length protein [60].

So how might these observations be reconciled? The caveat that the Deyaert study was not made on full length LRRK2 should again be stressed here, but fascinatingly, their data do point to an explanation. Specifically, their results suggest that pathogenic mutations may not actually decrease GTPase activity *per se*, but instead slow the monomerisation of GTP-bound RocCOR dimers, which their data indicate to be a prerequisite step before GTP hydrolysis [31]. As such, these pathogenic mutations can be expected to trap LRRK2 as GTP-bound dimers, which would be entirely consistent with elevated substrate phosphorylation.

**Fig. 2:**
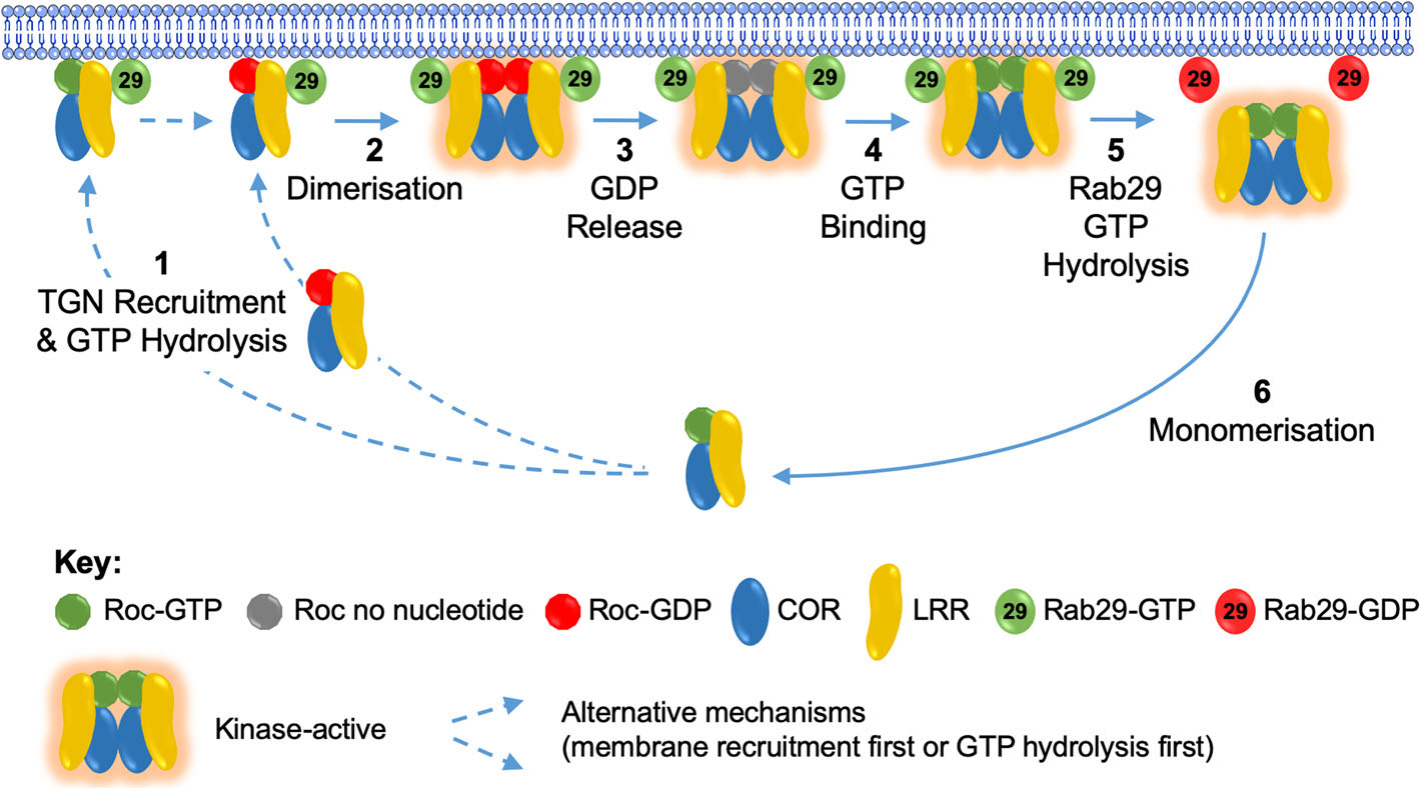
Rab29-dependent LRRK2 activation model. In the presence of GTP-bound Rab29 the equilibrium between monomeric cytosolic LRRK2 and kinase-active dimeric membrane-associated LRRK2 is shifted to the membrane form. **1** Monomeric LRRK2 is recruited to TGN membranes by GTP-bound Rab29. It is not known whether LRRK2 GTP hydrolysis occurs in the cytosol or immediately following membrane recruitment, but the result is an accumulation of monomeric GDP-bound LRRK2 on TGN membranes. **2** The recruitment of LRRK2 to TGN membranes creates a microdomain of high LRRK2 concentration, facilitating LRRK2 dimerisation. **3** While dimerised and kinase-active, LRRK2 releases GDP, **4** GTP exchange occurs, creating dimeric, kinase-active and Rab29-bound LRRK2. **5** Rab29 GTP hydrolysis releases LRRK2 dimers, promoting dissociation from TGN membranes. **6** Decreased LRRK2 concentration in the cytosol favours monomerisation and kinase inactivation. This last step is impaired by pathogenic RocCOR mutations. The representation of LRRK2 as LRR, Roc and COR domains is derived from the LRRK2 GTPase cycle proposed by Deyaert and colleagues, upon which much of this model is built (31).

Integrating this with LRRK2 kinase activation by membrane recruitment by active GTP-bound Rab29 yields the following theoretical model for LRRK2 activation, which is outlined in Fig. 2. Interactions between the ankyrin domain of LRRK2 and GTP-Rab29 leads to membrane recruitment of LRRK2 monomers, creating a microdomain of high LRRK2 concentration, which helps promote LRRK2 dimerisation. The combination of membrane localisation and dimerisation leads to Rab protein phosphorylation. GDP dissociates from LRRK2 to be replaced by GTP, favouring the dissociation of LRRK2 dimers, and the return of monomeric LRRK2 to the cytosol. However, binding to GTP-Rab29 might be expected to stabilise LRRK2 dimers, such that the release and monomerisation of LRRK2 could require Rab29 to hydrolyse its own bound guanine nucleotide and enter an inactive GDP-bound conformation. As such, Rab29 may enhance LRRK2 kinase activity in three ways: first, by recruiting LRRK2 to the subcellular localisation where its substrates are; second, by creating a LRRK2-rich microenvironment that favours dimerisation and increased kinase activity; and third, by stabilising LRRK2 dimers and preventing their monomerisation when GTP-bound. Pathogenic mutants synergise with Rab29 to further enhance substrate phosphorylation, by either further stabilising LRRK2 dimers (RocCOR mutants), or by elevating intrinsic LRRK2 kinase activity (G2019S).

These observations therefore point to a model where LRRK2 dimerisation promotes kinase activity, and the GTPase activity acts to determine the duration for which dimers exist. However, it must be noted that the relationship between LRRK2 GTPase and kinase activities and dimerisation is more complicated yet, since a number of autophosphorylation sites have been reported within the LRRK2 Roc domain. The effect of this autophosphorylation is poorly understood, but likely indicates a bi-directional relationship between these two enzymatic activities. (Curiously, the LRRK2 kinase domain is itself the site of a number of autophosphorylation sites, suggesting yet another layer of complexity.) In addition, how WD40 domain dimerisation integrates into this is another source of confusion. On the one hand loss of this domain prevents dimer formation and reduces the neurotoxicity of LRRK2 [62], but on the other hand, low resolution structural studies of dimeric full-length LRRK2 do not position the two WD40 domains in sufficiently close proximity to interact [53], while PD-associated WD40 domain variants that lie within the dimerisation interface weaken interaction between isolated WD40 domains [63]. Clearly, many key experiments remain to be performed.

### LRRK2 signalling

Beyond the signalling mechanisms involving LRRK2 mentioned above (phosphorylation of Rab proteins, activation of LRRK2 kinase activity by Rab29, and modulation of GTPase function by ARHGEF7, ArfGAP1, and RGS2), LRRK2 has been linked to a variety of different signal transduction pathways, which we summarise in this section. These include pathways that are relevant to all or most cell types, and others that are probably most important in immune cells, which likely indicates that LRRK2 has a number of signalling functions that may be both cell and context specific. Most fascinatingly, within these various roles there is little evidence of LRRK2 acting as kinase, with most studies suggesting a scaffolding function, likely exerting an indirect modulatory effect on signalling.

### Signalling upstream of LRRK2

In addition to autophosphorylation by its own kinase domain, LRRK2 is phosphorylated by other kinases on a number of residues (i.e. heterophosphorylation). Most interest has focussed on a series of serines between the ankyrin and leucine-rich repeat domains that appear to exert a key regulatory role on LRRK2. Kinases that have been reported to phosphorylate these LRRK2 residues are casein kinase 1α (CK1α) [64], the IκB family kinases IKKα, IKKβ, IKKε and TANK-binding kinase 1 (TBK1) [65], and protein kinase A (PKA) [66, 67], with dephosphorylation mediated by the protein phosphatase PP1 [68, 69] and induced by arsenite or hydrogen peroxide [69]. This range of kinases is perhaps surprising, since their typical modes of action are notably different. CK1α is considered a ubiquitous and constitutively active kinase, so LRRK2 phosphorylation mediated by this kinase is likely to be modulated only at the level of substrate availability, rather than by activation or inactivation of the upstream kinase. By contrast, the IκB family kinases are activated by a number of cytokines and are especially important in immune cells, with LRRK2 phosphorylation reported to be increased following treatment of macrophages with some, but not all, Toll-like receptor (TLR) agonists, and not by agonists of other immune relevant pathways [65]. Under physiological conditions, TLRs are activated by pathogens, forming part of the innate immune response, so these observations are perhaps more relevant for the role of LRRK2 in CD, TB and leprosy, although it is worth noting that these proteins are expressed throughout the brain (in particular in microglia) [70]. Finally, PKA is well established to be activated by the second messenger cyclic AMP (cAMP), the production of which is triggered by a variety of extracellular ligands and it is likely pertinent to all cell types, not least neurons. However, PKA-mediated phosphorylation of LRRK2 has only been reported in cells after treatment with the pharmacological agent forskolin [67], which activates adenylate cyclase to increase cAMP production, rather than after treatment with a physiological agonist. As such, the context under which PKA phosphorylates LRRK2 is not clear, although this story is supported by reports of an interaction between LRRK2 and the PKA regulatory subunit, RIIβ [71, 72].

What these phosphorylation events have in common, is their effect on the binding of LRRK2 to 14-3-3 proteins. 14-3-3 proteins are a family of seven highly conserved proteins that have been reported to bind hundreds of other proteins (the 14-3-3ζ isoform alone has over 500 unique interactors listed on *BioGRID* [73]). In the majority of cases, 14-3-3 proteins bind specifically to motifs containing phosphoserine or phosphothreonine, and thus protein-protein interactions involving 14-3-3 proteins are typically phosphorylation dependent [74]. This is certainly true for LRRK2, which was first reported to bind all 14-3-3 isoforms other than 14-3-3σ following phosphorylation of two LRRK2 residues, serine-910 and serine-935 [66, 75, 76]. 14-3-3 proteins have also been reported to bind LRRK2 via PKA-mediated phosphorylation of a serine residue (serine-1444) within the LRRK2 Roc domain [67]. The effect of the LRRK2-14-3-3 interaction is not fully established, but the evidence, largely from studies using LRRK2 constructs containing non-phosphorylatable serine-to-alanine amino acid substitutions at the relevant phosphorylation sites, suggests that 14-3-3 binding prevents the self-association of LRRK2 into dimers and perhaps higher order multimers, and thereby affects both the activity and subcellular localisation of LRRK2 [67, 69, 75]. Much work remains, but this interaction appears to be of relevance to PD, since co-expression of 14-3-3θ has been reported to rescue the decrease in neurite outgrowth seen in cultured neurons over-expressing the pathogenic LRRK2 variants, R1441G or G2019S [77].

### Signalling downstream of LRRK2

Over the years a great deal of work has gone into uncovering signalling pathways modulated by LRRK2. Initial work focussed on MAP kinase pathways, with some evidence found that LRRK2 may affect the activity of all four classical MAPK pathways: ERK1/2, ERK5, p38 MAPK, and JNK (reviewed by us in [39]). However the lack of follow-up studies suggest that any role for LRRK2 in these pathways is most likely subtle, for example controlling subcellular localisation of signalling components via protein-protein interactions, in particular with MKK3/6/7 and JIPs1-4 [78–80]. Nonetheless, interest in LRRK2 as a signalling protein has continued and this protein has been implicated in a variety of pathways, in addition to those we expand upon below. These also include, but are not limited to, Akt, Notch and FADD pathways [81–83].

In light of the relevance of LRRK2 to immune cells, it is unsurprising that a number of studies have connected this protein to activation of the transcription factor NF-κB, a classical mediator of inflammatory responses. However, the data are not clear cut, on the one hand agreeing that LRRK2 over-expression stimulates NF-κB activity [84, 85], while disagreeing on the effect of loss of LRRK2, with decreased NF-κB activity reported in Lrrk2 knockdown microglia [85], but increased activity found in microglia derived from Lrrk2 knockout animals [86]. Impaired NF-κB activation has been reported in both fibroblasts and iPSC-derived neurons from individuals carrying pathogenic LRRK2 mutations [87, 88]. There is clearly more work that needs to be carried out before a precise role for LRRK2 in this signalling mechanism can be determined, but two important observations can be made. First, even though NF-κB is typically activated by many of the same stimuli that trigger LRRK2 phosphorylation via IκB family kinases, LRRK2 does not appear to be required for the activation of NF-κB by the same TLR ligands that drive LRRK2 phosphorylation (i.e. TLR activation and the subsequent activation of NF-κB and LRRK2 phosphorylation are not part of a linear pathway). And second, certain data indicate that LRRK2 may exert an effect on NF-κB via an intriguing mechanism involving PKA, which in principle puts PKA both up and downstream of LRRK2, similarly to Rab29. In particular, recent data indicate that through an as yet undetermined mechanism LRRK2 represses phosphodiesterase 4, an enzyme responsible for cAMP degradation, leading to increased PKA activity and enhanced PKA-mediated phosphorylation of the NF-κB p50 subunit on an inhibitory phosphorylation site [71, 86]. Although this story is incomplete it could have relevance for neuronal biology and in particular PD, since LRRK2 has also been reported to reduce PKA activity induced by stimulation of the D1 dopamine receptor [72].

A large body of evidence implicates LRRK2 in intracellular calcium signalling. These include observations of altered mitochondrial and endoplasmic reticulum calcium signalling in pathogenic LRRK2 neuronal models [89–92], and roles for LRRK2 in modulating plasma membrane calcium channels [93, 94]. Given the well-established roles of calcium at neuronal synapses, these data suggest pathogenic LRRK2 mutations may affect synaptic physiology at least in part via an effect on intracellular calcium, which could have clear relevance to early stages of neurodegeneration in PD. Importantly, there is already data supporting this idea ([95, 96]. Furthermore, in addition to endoplasmic reticulum and mitochondria calcium signalling, LRRK2 has been implicated in calcium signalling at lysosomes, the third major intracellular calcium store [97, 98]. Lysosomes are degradative organelles that represent the end points of both endocytosis and autophagy, so this story could also be of great potential relevance to neurodegeneration. Evidence of roles for LRRK2 in these and other cell biological processes are summarised below.

Finally, in the context of LRRK2 function in immune cells, LRRK2 has been implicated in regulating the calcium activated transcription factor NFAT. NFAT is of central importance to the innate immune response [99], but also relevant to neuronal biology [100]. Under basal conditions NFAT is retained in the cytoplasm by an inhibitory NRON complex, but is activated via dephosphorylation by the calcium-activated phosphatase calcineurin, which allows NFAT to dissociate from the NRON complex and enter the nucleus, driving subsequent gene expression. Based on initial observations made in immune cells from an experimental model of CD, LRRK2 appears to form part of this complex, where it strengthens the repression of NFAT [99]. Correspondingly, loss of *Lrrk2* potentiates NFAT-dependent changes in gene expression induced by zymosan, a yeast cell wall component [99]. Since zymosan acts independently from the TLRs reported to elicit LRRK2 phosphorylation [65, 99], LRRK2 phosphorylation by IκB family kinases is unlikely to form part of this mechanism.

### Wnt signalling and GSK3

The connections between LRRK2 and Wnt signalling pathways, and more generally, signalling mechanisms involving the serine/threonine kinase GSK3β, exist both up and downstream of LRRK2 and are so broad that we will address them separately in this section.

Through interactions with multiple Wnt signalling proteins, LRRK2 has been linked to both the canonical/β-catenin and non-canonical/PCP Wnt signalling pathways [101]. Interactors include the membrane receptor LRP6 [102], key intermediary proteins such as DVL proteins [103, 104], Axin1 [102, 105], GSK3β [102, 105–107], PRICKLE1 and CELSR1 [104], and the canonical Wnt effector β-catenin [105]. LRRK2 appears to act as a scaffolding protein in these pathways, potentially exerting effects at multiple stages, such that over-expressed LRRK2 can cause apparently contradictory effects on β-catenin activation depending on which other Wnt signalling component it is over-expressed with. Nonetheless, the overall consensus is that LRRK2 represses canonical Wnt signalling [104, 105], while activating the non-canonical/PCP pathway [104]. Since these pathways are usually mutually antagonistic, this suggests LRRK2 may play a role in determining the balance between them [101, 104]. Importantly, PD-causing mutations throughout LRRK2 appear to further repress canonical Wnt signalling [46, 102, 105], although not all publications agree [104], while the protective LRRK2 variant R1398H has the opposite effect [46]. Given the well-established requirements for Wnt signalling pathways in the development of the brain and in particular the dopaminergic neurons of the ventral midbrain that are typically lost in PD [108], these observations implicate dysregulated Wnt signalling as a plausible mechanism underlying neurodegeneration caused by LRRK2 mutations.

However, altered canonical and non-canonical Wnt signalling is not the end of this story. As we describe below, LRRK2 is connected to microtubule biology via a number of studies, which include investigations into phosphorylation of the axonal microtubule-binding protein Tau [106, 107, 109–111]. Tau phosphorylation, which causes the detachment of this protein from microtubules in turn leading to the accumulation of neurofibrillary tangles and microtubule destabilisation, is a classical hallmark of Alzheimer’s disease [112]. Nonetheless, GWAS indicate that Tau is also highly relevant to PD [113], with post-mortem Tau pathology having been reported in brains from PD patients harbouring pathogenic LRRK2 mutations [4, 114, 115] and in LRRK2 mouse models [116–118]. Importantly, studies indicate that LRRK2 promotes Tau phosphorylation either directly [110], or, perhaps more plausibly, indirectly, by acting as a scaffold to enhance Tau phosphorylation by GSK3β, which is very well described as physiological Tau kinase [106, 107]. This latter mechanism is fascinating, since a suggested effect of LRRK2 in canonical Wnt signalling is to promote the inhibitory phosphorylation of β-catenin, again via a scaffolding effect on GSK3β [105]. In both cases, activation of canonical Wnt signalling relieves this phosphorylation [112]. As such, LRRK2 may contribute to both the Wnt-mediated control of β-catenin and Tau via a scaffolding action on GSK3β. Both these events have clear relevance for neurodegeneration. Taking this one step further, it is fascinating to note that the inhibitory phosphorylation of NFAT in the NRON complex is also mediated by GSK3β [99]. Thus, LRRK2 enhances GSK3β activity via a scaffolding action in three distinct protein complexes, and it is therefore temping to speculate that enhancement of GSK3β-mediated phosphorylation may turn out to be a key feature of LRRK2 signalling.

### Cell biological functions

**Fig. 3:**
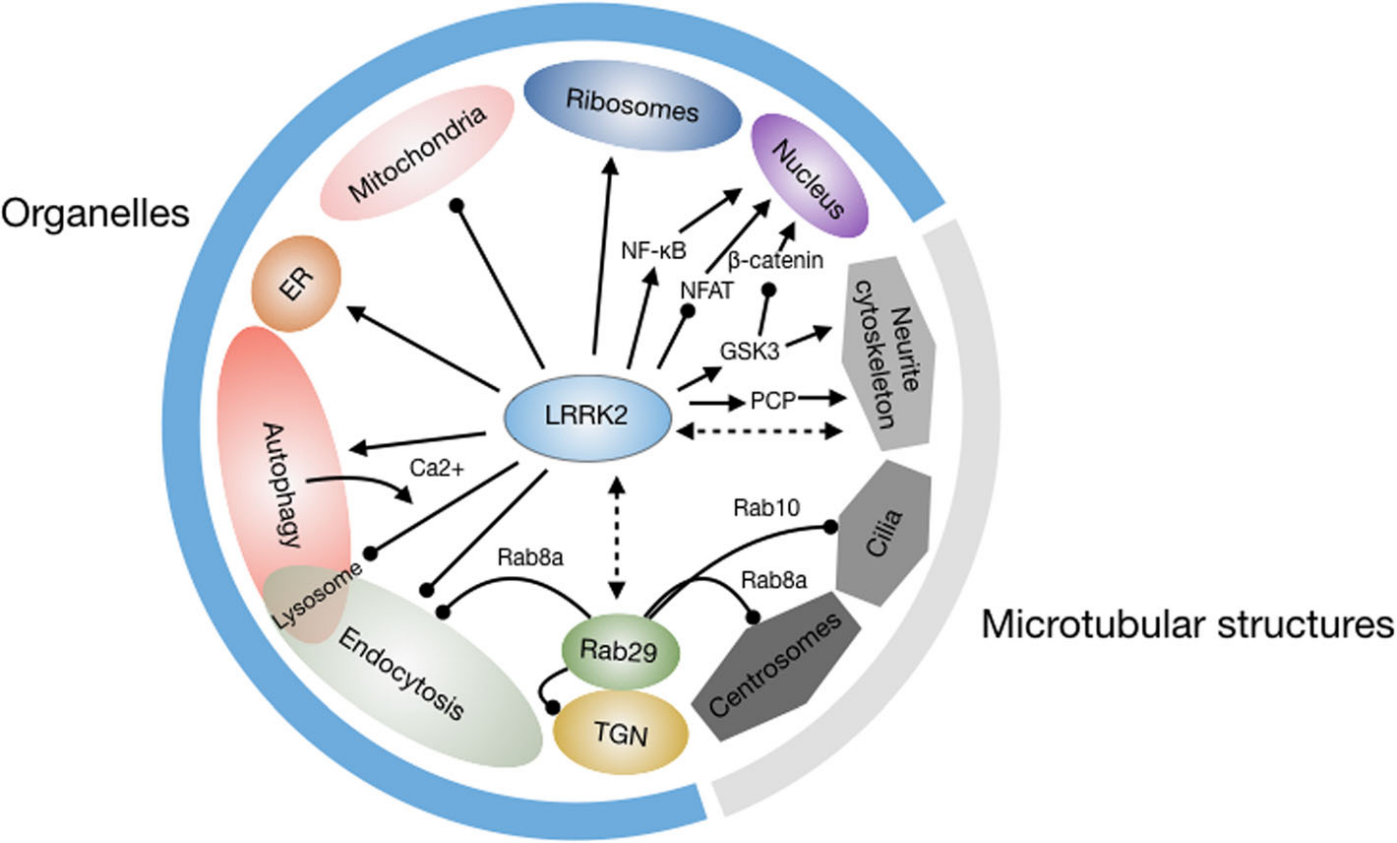
Cell biological processes impacted by LRRK2. As outlined in the main text, LRRK2 has been implicated in regulating processes at membranous organelles and microtubule-based structures, which are shown in the Figure, as are certain LRRK2-mediated signalling pathways that are likely to be involved. Although not mentioned in the main text, the nucleus is included as the subsequent organellar target of transcription factors affected by LRRK2 signalling. The direct interactions between LRRK2 and Rab29, and LRRK2 and microtubules, are depicted with dashed double-headed arrows. Regulatory mechanisms that are clearly inhibitory to the target are depicted with a round-headed arrow; all other relationships (whether activatory, too complex to categorise as inhibitory or activatory, or as yet undefined) are depicted with conventional arrows.

Shortly after the discovery of LRRK2, attempts to visualise the distribution of endogenous LRRK2 within the rodent brain using polyclonal antibodies revealed intense staining across membrane-bound organelles and vesicular structures, with greatest enrichment across substantia nigra, thalamus and in particular striatal areas [119, 120]. Although subsequent studies have demonstrated these antibodies lack specificity [121], work in cellular models has corroborated the observations, leading to strong claims of roles for LRRK2 in endocytosis and autophagy [44, 122–124]. In the rest of this review, and as summarised in Figure 3, we discuss roles for LRRK2 in these and other cell biological processes where a convincing body of evidence has been established.

### Autophagy

Autophagy (from the ancient Greek, “self-eating”) is a highly specialised mechanism to ensure homeostasis through degradation of unwanted cellular components. Three major types of autophagy have been described; microautophagy, chaperone-mediated autophagy and macroautophagy [125].

Studies using LRRK2 knockout mice, which curiously display their most striking phenotype in the kidney [126], leave little doubt that loss of LRRK2 impacts upon macroautophagy, but shed little light on what role(s) LRRK2 plays in this process. Within the kidneys of these animals, bi-phasic age-dependant alterations in macroautophagic activity have been reported. These organs display increased macroautophagic activity at 7 months, as determined by expression of the macroautophagy markers LC3-II and p62 and the accumulation of lipofuscin and α-synuclein, and decreased macroautophagy at 20 months [127]. Increased expression of lysosomal enzymes, perhaps beginning shortly after birth, was also reported – an observation that has been confirmed independently [127–129]. Why loss of LRRK2, a protein known to mediate profound neurological phenotypes in humans, manifests in the kidney, is not immediately clear. One possible explanation may lie with the high levels of LRRK2 in the kidney and the comparatively low levels of homologous LRRK1, which may fulfil a compensatory role in other tissues [127]. In support of this hypothesis, generation of double LRRK knockout mice appear to recapitulate kidney autophagy defects in the brain [130].

Mechanistic investigations into LRRK2 and macroautophagy performed in cellular models similarly leave little doubt that LRRK2 is involved in the process, but often disagree about what that role might be (reviewed in [131]). That said, it seems likely that the relationship is both cell type-specific and complex, with LRRK2 perhaps modulating early and late steps of the macroautophagy pathway (i.e. macroautophagy induction and lysosomal function). As judged by levels of the autophagic marker LC3-II, pharmacological inhibition of endogenous LRRK2 kinase activity stimulates autophagy in H4 neuroglioma cells, SH-SY5Y neuroblastoma cells, HEK293T cells and primary astrocytes [132–134], but decreases this process in RAW264.7 macrophages and BV2 microglia, which are both monocyte cell lines [135]. The knockdown or knockout of LRRK2 also appears to have differential effects depending on the model used [133–135]. Furthermore, rapamycin-induced autophagy causes a higher recruitment of LRRK2 to membrane fractions containing the autophagic markers LC3-II, ATG7 and CathepsinD in monocytes, which suggests a role in mTOR-dependent autophagy [135], whilst LRRK2 kinase-dependent regulation of LC3-II in H4 neuroglioma cells is independent of mTOR [134]. Regarding pathogenic LRRK2 mutations, a number of early studies report that over-expression of wildtype or mutant LRRK2 induces autophagy [97, 136, 137], yet primary neurons derived from G2019S knock-in mice show reduced autophagic activity [116], and human fibroblasts derived from pathogenic LRRK2 mutation carriers show a consistent reduction in starvation-induced macroautophagy [138]. When taken together it is fair to conclude that LRRK2 is very likely involved in macroautophagy, but clearly further work is required before any consensus role can be established.

Finally, in addition to regulating macroautophagy, LRRK2 may itself be a substrate of autophagy, although in this case, chaperone mediated autophagy (CMA). CMA is a selective process where protein substrates are trafficked across the lysosomal membrane via interactions with Heatshock cognate 70, which in turn binds the lysosomal receptor, LAMP2A [139]. In studies using both *in vitro* cell lines and brain slices, treatment with lysosomal inhibitors caused an increase in intracellular LRRK2 [140]. Interestingly, the pathogenic LRRK2 G2019S variant was not as efficiently degraded as its wildtype counterpart whilst overexpression of either LRRK2 construct was sufficient to inhibit CMA. As such, the study suggests a mechanism where pathogenic forms of LRRK2 that are inefficiently turned over by CMA cause an inhibition of CMA that leads to the accumulation of other CMA substrates [140].

### Endocytosis

Several lines of evidence point to LRRK2 as a modulator of endocytosis, including endocytosis of synaptic vesicles in neurons. Endocytosis is the process of internalisation of membrane associated proteins via a series of organellar structures, with the final destination either being the lysosome for degradation or recycling into other intracellular locations. Studies linking LRRK2 and endocytosis include the identification of LRRK2 as an interacting partner of Rab5b at neuronal synapses [141]. Rab5 protein acts as a regulator of trafficking and fusion of endocytic vesicles from plasma membrane to early endosomal compartments, i.e. early stages of endocytosis [141, 142]. Both depletion and overexpression of LRRK2 impaired the rate of synaptic vesicle endocytosis and was rescued by co-expression with Rab5b [141]. The same group further suggested Rab5b is directly phosphorylated by LRRK2, causing it to exhibit stronger GTPase binding activity [143]. Although it is important to note that phosphorylation is at a different residue to the conserved phosphorylation site in other Rab proteins, others studies do not support Rab5b as a LRRK2 substrate [35]. In any case, the use of a phosphomimetic Rab5b mutant indicated that Rab5b phosphorylation acts as a negative regulator of neurite outgrowth. This could also be relevant beyond neuronal cells, as this construct also impaired EGF receptor (EGFR) degradation in HeLa cells [143], which is consistent with slower endocytosis.

In contrast to Rab5 proteins, Rab7 acts at later stages in endocytosis, including at lysosomes. Rab7 has been connected to LRRK2 in a small number of papers, the first being a report of a physical interaction between these proteins in *Drosophila* [144]. Rates of endocytosis were not investigated, although an effect on lysosomal positioning was reported [144]. Nonetheless, two subsequent papers indicate that LRRK2, and to a greater extent, pathogenic forms of LRRK2, slow EGFR degradation by impairing later stages of endocytosis and that Rab7 is involved in this process [145, 146]. Together these studies find a fascinating regulatory model where LRRK2-mediated phosphorylation of Rab8A leads (through an as yet undefined mechanism) to decreased Rab7 activity, which leads to EGFRs failing to traffic to lysosomes and instead accumulating in a Rab4-containing vesicular compartment [146]. The consequences of delayed endocytosis in this context have not been established, but since receptor internalisation is intrinsically linked to strength and duration of signalling pathway activation [39], this could have implications for cascades relevant to cell survival.

Returning to endocytosis in neurons, down-regulation of synaptic vesicle endocytosis in the absence of LRRK2 has also been corroborated in a number of studies [95, 147–150]. For example, LRRK2 knockdown in cortical neurons caused redistribution of synaptic vesicles to the recycling pool and fewer docked vesicles in contact with the presynaptic membrane [95]. Furthermore, several of these findings have been replicated following treatment of wildtype neurons with LRRK2 inhibitors, suggesting a kinase-dependent mechanism [147].

One suggested mechanism of action is EndophilinA phosphorylation [148, 149, 151]. Similarly to several membrane associated proteins, EndophilinA contains a BAR domain that is believed to modulate membrane curvature and vesicle release. In the first of these papers the authors reported that phosphorylation of the Endophilin A BAR domain by LRRK2 hampers its ability to dissociate from membranes causing membrane deformation and impaired synaptic vesicle endocytosis at *Drosophila* neuromuscular junctions [148]. Fascinatingly, both hyperphosphorylation elicited by over-expression of the LRRK2 G2019S variant or loss of basal EndophilinA phosphorylation following knockout of the *Drosophila* LRRK2 orthologue caused a similar impairment of synaptic vesicle endocytosis [148]. These data suggest a requirement for tight regulation of EndophilinA phosphorylation for normal neuronal functioning, and are also consistent with the previous observation of impairments in synaptic vesicle endocytosis following knockout or overexpression of LRRK2 [141]. In a further twist, LRRK2-dependent EndophilinA phosphorylation has also been implicated in neuronal autophagy, with phosphorylated EndophilinA reported to recruit the autophagic factor Atg3 during induced autophagosome formation [151]. The relevance of EndophilinA phosphorylation to mammals remains to be established, although LRRK2 can phosphorylate human EndophilinA proteins *in vitro* [149]. Nonetheless EndophilinA phosphorylation is an interesting story to follow.

LRRK2 has also been implicated in other synaptic vesicular trafficking processes and in behavioural phenotypes that are consistent with altered synaptic properties, although these studies have produced a range of results, perhaps a consequence of the different neuronal populations and models studied. Overexpression of wildtype LRRK2 in BAC-transgenic mice showed elevated release of striatal dopamine, whereas G2019S mice showed an age-dependent decrease in striatal dopamine uptake, release and content [152]. A separate study further reported D2-receptor mediated short-term plasticity defects in striatal glutamate neurons of mice overexpressing wildtype LRRK2 [153]. Interestingly, no synaptic abnormalities have been reported in the striatum of LRRK2 knockout mice [153]. Behavioural analysis of wildtype LRRK2 overexpression models has yielded conflicting results with reports of both hyperactivity and enhanced motor performance and hypoactivity and memory impairments [152, 153]. Overexpression of G2019S-LRRK2 has been shown to cause deficits in long term depression and age-dependent dysfunctional plasticity in the hippocampus [154].

Fascinatingly, LRRK2 is linked to neuronal endocytic events involving proteins encoded by genes implicated in autosomal recessive early-onset parkinsonism: *SYNJ1* [155, 156] and *DNAJC6* [157]. *SYNJ1* encodes for the synaptically enriched lipid phosphatase Synaptojanin1 which functions in the uncoating of neuronal vesicles. Similarly, the neuronal specific protein Auxillin, encoded by *DNAJC6*, acts as a co-chaperone with Hsc70 to uncoat clathrin vesicles [158]. Intriguingly, both of these proteins have recently been nominated as direct targets of LRRK2 phosphorylation [150, 159]. Elevated synaptojanin1 phosphorylation was first observed in *Drosophila* modified to express human R1441C [160]. The direct phosphorylation of synaptojanin1 by LRRK2 was subsequently demonstrated *in vitro* and shown to impair interactions with endophilinA [150]. LRRK2 phosphorylation of auxilin was similarly found to disrupt interactions with clathrin, resulting in endocytic defects and decreased synaptic vesicles in patient-derived iPSC dopaminergic neurons [159]. These results await independent replication but the fact these three PD-related genes – *LRRK2*, *SYNJ1* and *DNAJC6* – might act in a common pathway is clearly intriguing. Moreover, these observations form part of a wider body of data connecting PD with endocytosis and membrane trafficking processes more generally, for example reviewed by [161]. The strength of this connection is underscored by recent evidence that genes encoding endocytosis proteins contribute significantly to the polygenic risk of PD [162].

Lastly, it is worth stressing that both autophagy and endocytosis terminate in lysosomes, which strongly suggests these two processes impinge upon each other. With this in mind we note a recent study describing an intersection of LRRK2 with both upstream and downstream Rabs at lysosomes [124]. Treatment of cells with the lysosomal stressor chloroquine induced LRRK2 recruitment onto enlarged lysosomes with co-expression of Rab29 facilitating this phenotype. Overexpression screening of 27 different Rab GTPases revealed the LRRK2 substrates Rab8a and Rab10 colocalising with LRRK2-positive lysosomes under stressed conditions in a LRRK2 kinase dependent manner [124]. Interestingly, Rab29 activation of LRRK2 reduced stress-associated lysosomal enlargement and upregulated lysosomal secretion, whereas Rab8a suppressed enlargement and Rab10 promoted secretion. Taken together, these observations support a model whereby Rab29, LRRK2 and its Rab substrates participate in lysosomal homeostasis [124].

### Trans-Golgi Network

Evidence connecting LRRK2 to Rab29 has also linked LRRK2 to a category of organelle that is not directly part of the endocytic or autophagic pathways: the *trans-*Golgi network (TGN). In the first of these publications, overexpression of the pathogenic G2019S variant was found to phenocopy Rab29 knockdown in significantly reducing mannose 6-phosphate receptor (MPR) localisation at the Golgi [41]. These were fascinating observations, since reduced MPR localisation at the TGN is a well described consequence of loss of retromer complex function [163]. The retromer complex mediates recycling of transmembrane receptors from endosomes towards the TGN, and a key component of this complex is VPS35. Remarkably, like *LRRK2* and *RAB29* variants, *VPS35* mutations are a cause of PD [164, 165], and in agreement, a PD-associated VPS35 variant, D620N, elicited a similar effect on MPR localisation [41]. The authors reported that LRRK2 physically interacts with VPS35, whilst overexpression of wildtype VPS35 rescues defects caused by mutant LRRK2 or Rab29 knockdown. Thus, their data suggests LRRK2 acts as a modifier of VPS35 function in recycling of proteins and membranes from the endosomal system to the TGN [41].

As mentioned, the second paper linking LRRK2 to Rab29 did so as part of a screen for LRRK2-interacting proteins, which also identified BCL2-associated athanogene 5 (BAG5) and Cyclin-G–associated kinase (GAK) as interactors [42]. In the context of PD, BAG5 and GAK are interesting proteins. *GAK* has been identified previously as candidate risk loci for sporadic PD by GWAS [41, 42], whereas BAG5 has been reported to promote degeneration of dopaminergic neurons through inhibition of the E3 ubiquitin ligase Parkin, which causes recessive PD [166, 167]. LRRK2, GAK, BAG5 and Rab29 were found to form a single complex revealed by fluorescence cell imaging to localise to the TGN where they promote a Golgi clustering phenotype [42]. Golgi clustering was enhanced by all known pathogenic variants of LRRK2, and the clustering induced by the overexpression of anyone of the four proteins could be at least partially rescued by knockdown of any one of the other three proteins. Fascinatingly, Golgi clustering appeared to require autophagy, as the effect of LRRK2 overexpression could also be rescued by inhibition of lysosomal acidification or knockdown of the key autophagy protein Atg7 [42]. As such, both studies demonstrated a physical interaction between Rab29 and LRRK2 that is of functional relevance to the Golgi, and in particular TGN, yet they differ regarding the effects of Rab29 manipulation. Some of these differences can be ascribed to the different experiments performed, yet both studies performed comparable neurite outgrowth assays. Confusingly, Beilina *et al.* find that knockdown of Rab29 rescues the decreased neurite outgrowth caused by LRRK2 overexpression and overexpression of Rab29 replicates the phenotype [42], whereas MacLeod *et al.* report that decreased neurite outgrowth elicited by LRRK2-G2019S is rescued by overexpression of Rab29 and replicated by Rab29 knockdown [41]. As such, the two papers disagree entirely on whether greater Rab29 activity is beneficial or detrimental to cells.

Subsequent findings favour the idea that elevated Rab29 activity is cytotoxic. Notably, Rab29 recruitment of LRRK2 to the TGN enhances LRRK2 kinase activity [43, 44]. Since increased LRRK2 kinase activity appears to be a close correlate of LRRK2 pathogenicity, this is clearly consistent with Rab29 activity being detrimental to cells. Importantly, the clustered Golgi phenotype observed following LRRK2-Rab29 colocalisation has been replicated [43, 44]. This exciting story is still evolving, but additional details include roles for CK1α and guanosine-nucleotide exchange factor, ARHGEF7 in regulating recruitment of LRRK2 to the TGN [64]. As mentioned above (see also Fig. 1), CK1α is a kinase responsible for constitutive phosphorylation of LRRK2 [64], whereas ARHGEF7 has previously been reported to modulate LRRK2 GTPase activity [51].

### Mitochondria

The mitochondria – organelles responsible for cellular respiration and energy production – are heavily implicated in PD, with numerous lines of evidence demonstrating that mitochondrial dysfunction is part of the pathology of idiopathic PD and certain types of familial PD. Most notably, decreased mitochondrial complex 1 activity in the *substantiae nigrae* of PD patients is a well-established phenomenon [168], and a number of environmental toxins that cause nigral legions and parkinsonian phenotypes in humans and animal models are inhibitors of this aspect of mitochondrial function [169–171]. Furthermore, proteins encoded by several genes associated with early-onset forms of familial PD, such as *PINK1* and *Parkin*, function within a common evolutionarily conserved pathway responsible for removing dysfunctional mitochondria by mitophagy (reviewed by others, e.g. [172, 173]). Loss of these proteins causes an accumulation of larger mitochondria, decreased ATP production and increased levels of reactive oxygen species, leading to a selective loss of dopaminergic neurons of the *substantia nigra*. It is worth noting that since the cell loss caused by *PINK1* or *Parkin* mutations is so selective and not usually accompanied by Lewy body formation they may not represent a true form of PD but ‘nigral mitochondrial cytopathies’ [174]. In any case, it is evident that the group of dopaminergic neurons lost specifically in PD are especially sensitive to mitochondrial dysfunction, so any implication of LRRK2 in mitochondrial biology is of great interest.

When taken together, the evidence that pathogenic LRRK2 variants cause an impairment in mitochondrial function is very strong. For example, in *Drosophila* and transgenic mice, the G2019S LRRK2 variant has been shown to impart an increased sensitivity to mitochondrial toxins [175, 176], with a similar response reported in dopaminergic neurons derived from *LRRK2* mutation carriers [177, 178]. Nonetheless, it remains to be established whether pathogenic forms of LRRK2 cause an underlying defect in mitochondrial function that makes these organelles more sensitive to toxins, or whether LRRK2 mutants reduce the cell’s ability to cope with damaged mitochondria. These mechanisms are not mutually exclusive and there is data supporting either possibility. For example, LRRK2 G2019S is reported to increase phosphorylation of peroxiredoxin-3 (PRDX3), a scavenger of hydrogen peroxide produced by mitochondria, causing inhibition of endogenous peroxidases [179, 180]. But on the other hand, a detailed study of primary human fibroblasts from both manifesting and non-manifesting *LRRK2* mutation carriers, revealed a convincing preclinical impairment in the activities of mitochondrial complexes III and IV [181]. Supporting this interpretation further, a number of studies report that *LRRK2* mutations elicit abnormalities in mitochondrial morphology – in particular mitochondrial fragmentation [182–184].

It is interesting to note that this weight of evidence linking LRRK2 and mitochondria functionally is not matched by a corresponding amount of data supporting a physical interaction. Early investigations did report LRRK2 localisation on mitochondrial membranes [119, 185], but these observations have not been replicated to the extent that might be expected. As such, any association between LRRK2 and this organelle is most likely transient and/or context specific. In agreement with this, there is evidence that wildtype LRRK2 is recruited to the mitochondrial outer membrane in human iPSC-derived neurons treated with the mitochondrial depolarising agents Antimycin A and CCCP [186]. Therefore, if LRRK2 is not permanently located on mitochondria, how might pathogenic LRRK2 mutations cause defects in mitochondrial morphology and respiration? One possibility is via altered mitochondrial biogenesis [182], nonetheless we note that the reported recruitment of LRRK2 to the mitochondrial outer membrane induced by mitochondrial depolarisation involves a physical interaction between LRRK2 and a protein called Miro [186]. Miro is an outer mitochondrial membrane protein that acts as a tether, attaching mitochondria to microtubule motor proteins, thereby facilitating the transport of mitochondria along microtubules. The authors further reported that the binding of LRRK2 to Miro triggers transport of damaged mitochondria along axonal microtubules, leading to their removal by mitophagy (a type of macroautophagy that is selective for damaged mitochondria) [186]. Fascinatingly, this response is lost in cells expressing LRRK2 G2019S, as this pathogenic variant is not recruited to Miro by mitochondrial depolarisation [186]. The result of this is a reduction in the removal of damaged mitochondria by mitophagy. Intriguingly, these observations are consistent with independent studies reporting that pathogenic LRRK2 RocCOR domain mutations also elicit decreased rates of microtubule-based mitochondrial trafficking in axons [187](188]. Since LRRK2 is heavily linked to microtubules, we return to these cytoskeletal structures later in this article. Nonetheless, these are exciting observations, suggesting a pathomechanism at the crossroads of three important aspects of LRRK2 biology: microtubules, macroautophagy and mitochondria.

### Endoplasmic reticulum

Another intracellular organelle to which LRRK2 has been linked is the Endoplasmic reticulum (ER). This organelle is responsible for the translation, folding and trafficking of newly synthesised membrane and secreted proteins, and is also the major store of intracellular calcium. In the event of protein misfolding, the accumulation of misfolded proteins within the ER lumen can trigger a stress response that halts protein translation and activates signalling pathways to increase production of molecular chaperones [189]. An elevated ER stress response is well described in PD brains and may represent a protective mechanism to restore protein homeostasis [190]. Given the range of processes linked to LRRK2 it is perhaps unsurprising that LRRK2 has been implicated in ER stress responses, first indirectly, via the localisation of LRRK2 to the ER of intact dopaminergic neurons in healthy and PD brain tissue [191], and also directly, as part of a possible protective mechanism [192]. In particular, LRRK2 expression was found to be required for the full upregulation of GRP78, an ER chaperone protein, in SH-SY5Y cells treated with the ER stress inducing agent tunicamycin, an observation that correlated with increased cell death. Suggesting this may be of relevance to neurodegeneration, a similar requirement for LRRK2 was found for the induction of GRP78 expression and cell survival in response to the neurotoxin 6-hydroxydopamine [192]. This story is in its infancy, but it is interesting to note that the authors also found a similar effect on the resistance of dopaminergic neurons to 6-hydroxydopamine in nematodes lacking the LRRK2 ortholog, and provide evidence that similar mechanisms may operate in response to treatment with human α-synuclein [192].

In addition, LRRK2-mediated anterograde trafficking of vesicles from the ER to the Golgi has been described. This study found that LRRK2 selectively recruits Sec16A to the ER where it forms vesicle exit sites. As such, LRRK2 knockdown disrupted the association of Sec16 with exit sites and the transport of vesicles to the Golgi. Similarly, knock-in of the pathogenic LRRK2 variant R1441C weakened interaction with Sec16 leading to impairments of ER vesicle release [193].

### Ribosomes and translational control

LRRK2 has long been implicated in translational control via the direct phosphorylation of the protein synthesis machinery. This story began with a report that eukaryotic initiation factor 4E binding protein (4E-BP), a repressor of translation, is a substrate of the *Drosophila* LRRK2 homologue dlrrk [194]. This phosphorylation event was reported to inactivate 4E-BP thereby promoting cap-dependent protein synthesis, with prolonged phosphorylation leading to deregulated bulk protein translation [194]. Perhaps consistent with this, an independent study also using *Drosophila* reported an effect of postsynaptic expression of LRRK2 transgenes on presynaptic neurotransmitter release that can be blocked with protein synthesis inhibition [195]. However, other studies indicate that phosphorylation of 4E-BP by LRRK2 does not happen in mammalian systems. In particular, LRRK2 does not alter the phosphorylation of the human 4E-BP homologue in cells [196–198], and only phosphorylates this protein to a very low stoichiometry *in vitro* [197]. Nonetheless, LRRK2 has been reported to phosphorylate three human ribosomal proteins *in vitro* – S11, S15, and S27 – albeit also at low stoichiometry, but with S15 phosphorylation also observed in both mammalian neurons and *Drosophila* [198]. This paper produced two observations that are pertinent here. First, the authors were unable to demonstrate an effect of LRRK2 on 4E-BP phosphorylation in *Drosophila*, but were able to replicate the positive effect of LRRK2 on protein synthesis and extend this observation to mammalian neurons, although in this case the increase involved both cap-dependent and cap-independent translation [198]. And second, overexpression of a phosphodeficient S15 mutant protein (i.e. S15 with the LRRK2 phosphorylation site mutated to alanine) reduced the toxicity induced by pathogenic LRRK2 variants in *Drosophila*, rat and human neuronal systems [198]. Taken together, these studies suggest that 4E-BP most likely is not a LRRK2 substrate, but indicate that LRRK2 may yet exert a positive effect on protein synthesis in *Drosophila*. Whether this proves to be reproducible in mammals – and whether this involves phosphorylation of ribosomal proteins – remains to be seen, but given its potential importance, follow-up studies are eagerly awaited.

In addition to the above, LRRK2 is also reported to affect *Drosophila* gene expression at the post-transcriptional level via effects on microRNAs (miRNAs). miRNAs are considered to prevent translation by binding to mRNA molecules and blocking protein synthesis, while also destabilising the mRNA. In particular, LRRK2 was found to interact with Argonaute (Ago1), a component of the miRNA pathway [199]. In the brains of aged *Drosophila*, pathogenic human LRRK2 variants caused a down-regulation of dAgo1, which in turn led to a suppression of the activity of two miRNAs, let-7 and miR-184, and a corresponding increase in the translation of two target genes, DP1 and E2F1 [199]. Since DP1 and E2F1 are involved in the cell cycle, this suggested a potential pathomechanism whereby LRRK2 elicits overexpression of these proteins, which are likely toxic to post-mitotic neurons. Consistently, overexpression of either miRNA rescued the effect of overexpressed pathogenic LRRK2 variants on dopaminergic cell death, while partial loss of DP1 or E2F1 had similar effects [199]. Although this is a single study, the proposed mechanism via Ago1 creates the possibility that LRRK2 might alter the expression of multiple different miRNAs in different systems, so this is an intriguing story.

### Microtubule binding and cytoskeletal dynamics

In addition to cell biological processes involving membranous organelles, LRRK2 is strongly linked to the cytoskeleton, and in particular, microtubules. Microtubules are ubiquitous multimeric protein scaffolds that are central to many cellular structures and underpin a range of key cellular processes. As we outline in this section, LRRK2 has been implicated in a number of these: neurite outgrowth, trafficking of membranous cargo along axons, and the formation of cilia and centrosomes. Indeed, the inhibitory effect of LRRK2 overexpression on neurite outgrowth, which is potentiated by pathogenic mutations and has been replicated in multiple studies, is likely underpinned, at least in part, by binding of LRRK2 to microtubules (reviewed by ourselves elsewhere [200, 201]).

Interactions between LRRK2 and microtubules were first described in 2006 [119, 202], with binding eventually shown to be direct and specific to three β-tubulin isoforms, TUBB, TUBB4 and TUBB6 [61]. β-tubulins associate with related α-tubulins and the resulting heterodimers assemble into the long tubular structures that comprise microtubules. The LRRK2 binding site in β-tubulins was mapped to residues that are proximal to the binding site for the microtubule stabilising drug Taxol [61]. This suggested that LRRK2 binding may influence microtubule stability and consistently, LRRK2 knockout MEF cells displayed a significant increase in microtubule acetylation – a post-translational modification known to induce microtubule stability [203] – with similar observations subsequently made in the LRRK2 knockout mouse kidney [61, 129]. In addition, LRRK2 displayed a stronger localisation to microtubules in neuronal growth cones compared to those in adjacent axons, indicating a preferential binding to dynamic, i.e. not acetylated and stabilised, microtubules [61]. This prediction was confirmed in an independent study reporting that treatment of cells with deacetylase inhibitors or the tubulin acetylase αTAT1, both of which can be expected to increase tubulin acetylation, reduce the association of wildtype and pathogenic forms of LRRK2 with microtubule structures [187]. Fascinatingly, this latter study not only reported a stronger association between acetylated microtubules and the pathogenic LRRK2 RocCOR variants R1441C and Y1699C, but also, as mentioned above, found that these variants caused decreased transport of mitochondria along axonal microtubules in cortical neurons [187]. Perhaps surprisingly, the G2019S kinase domain mutant did not exert the same effect, suggesting this phenotype may be specific for LRRK2 GTPase mutations. Nonetheless, these observations of impaired mitochondrial trafficking underscore a key effect of altered microtubule function: disruption of microtubule-dependent organelle and vesicle transport. Disrupted trafficking of these membranous structures can cause subsequent deficits in processes they mediate (e.g. endocytosis, autophagy etc) and illustrate how microtubule integrity is vital for normal healthy cells.

Following the recent identification of Rab GTPase phosphorylation by LRRK2 [34], several groups have investigated downstream effects of elevated Rab protein phosphorylation, a path that has ultimately led to cell biological processes with microtubules at their core. In the first of such studies, phosphorylated forms of Rab8a and Rab10 were found to bind preferentially to two related proteins, Rab interacting lysosomal protein like 1 and 2 (RILPL1 and RILPL2) [35]. Rab8A, Rab10 and RILPL1/2 have all previously been reported to regulate primary ciliogenesis (i.e. the generation of primary cilia), suggesting that LRRK2 might also modulate this process. Primary cilia are microtubular projections emanating from the cell surface of numerous mammalian cell types and are considered key regulators of many signalling pathways. Fascinatingly, these include a number of cascades relevant to LRRK2, including Wnt and calcium signalling [204]. In any case, a role for LRRK2 in regulating ciliogenesis was confirmed as fibroblasts derived from knock-in mice carrying the pathogenic R1441G LRRK2 mutation displayed reduced starvation-induced cilia formation [35]. In subsequent work, cilia defects were also observed in fibroblasts and iPSCs derived from G2019S carriers, as well as striatal cholinergic neurons from a LRRK2 R1441C mouse model, demonstrating that multiple pathogenic LRRK2 mutations can impact on cilia formation. Critically, LRRK2 kinase inhibition was able to rescue defects caused by pathogenic LRRK2 both on cilia formation and cilia-dependent Sonic Hedgehog (Shh) signalling [205]. As such, the authors suggest mutant LRRK2 causes loss of cilia that may disrupt the ability of dopaminergic neurons to respond to neuroprotective Shh signals – an intriguing explanation of nigral legions in LRRK2-PD. The authors also reported that LRRK2 impairs ciliogenesis via the phosphorylation of Rab10 and not of Rab8a; fascinatingly, they found Rab8a and Rab10 to have opposing effects on cilia formation [205]. As such, it is plausible that LRRK2 might enhance ciliogenesis in cells where expression of Rab8a is high compared to that of Rab10.

The second microtubular structure linked to LRRK2 by Rab protein phosphorylation is the centrosome. Centrosomes are the primary microtubule organising centre in most cells, and are central to determining cell shape and polarity, as well as the positioning of spindle poles during mitosis. Perhaps very pertinently, centrosomes are structurally similar to cilia [206]. Two recent publications from Sabine Hilfiker’s laboratory link LRRK2 phosphorylation of Rab8a to centrosomal deficits [36, 207]. In the first paper, defects in centrosomal positioning and migration that were induced only by pathogenic LRRK2 variants and not wildtype LRRK2 were closely associated with an accumulation of pericentrosomal phosphorylated Rab8a [36]. This phenotype was rescued by expression of a non-phosphorylatable Rab8a mutant or by depletion of endogenous Rab8a. Intriguingly, a split centrosome phenotype was also observed within fibroblasts derived from LRRK2-G2019S carriers which was reversed by LRRK2-kinase inhibitors [36]. The second paper found that when recruited to the TGN by overexpressed Rab29, wildtype LRRK2 was able to induce the same phenotype via enhanced Rab8a phosphorylation [207]. Fascinatingly, whilst wildtype LRRK2 required both Rab29 and Golgi integrity to elicit this effect, pathogenic forms did not, indicating that activation of wildtype LRRK2 by Rab29 on TGN membranes may be a physiological mechanism that PD-causing variants are able to by-pass.

### Integrating the information: implications for PD

Since the discovery of *LRRK2* mutations as a cause of PD that is clinically indistinguishable from idiopathic PD, it has been hoped that mutant LRRK2 pathomechanisms will shed light on PD and perhaps create therapeutic targets to stop this condition at its earliest stages. As such, how the perturbed cell biological processes, that we have outlined above, might ultimately elicit neurodegeneration is perhaps the most important question remaining. This review summarises the basic biochemistry and cell biology of LRRK2, so a detailed commentary is clearly beyond the scope, but in this section, we nonetheless attempt to extract some pertinent observations from the mass of data. We would not expect these observations to guide future studies – our colleagues working on more translatable areas of LRRK2 research are well ahead of us here – but at the least we hope to provide some sort of context and direction for the reader.

So, which of the organelles and cell biological processes impacted by LRRK2 are most relevant? Let’s start from first principles. PD is ultimately a disease that is caused by dopaminergic cell death and is accompanied by the formation of Lewy bodies. Therefore, the most relevant effects of *LRRK2* mutations are clearly those that most easily account for these phenomena. Similarly, using an Ockham’s razor type of ‘simplest is most likely’ principle, we would also expect pathogenic *LRRK2* mutations to act in a common mechanism with PD-causing mutations in other loci. With this in mind, it is worth highlighting research into the molecular mechanisms by which *GBA* mutations elicit a similar type of late-onset PD to *LRRK2* (and indeed, to idiopathic PD). In particular, loss-of-function *GBA* mutations are now well established to cause lysosomal dysfunction, leading to impaired autophagic clearance of α-synuclein and a positive feedback loop where elevated α-synuclein causes a further impairment of lysosomal function [208]. Pathogenic mutations in *ATP12A2* appear to operate via a similar mechanism [208]. As such, impaired lysosomal function seems sufficient to account for the formation of Lewy bodies, which are primarily composed of α-synuclein.

As we have described, LRRK2 is connected to lysosomal biology via a number of mechanisms including endocytosis and autophagy, which both terminate with fusion of vesicular structures to lysosomes, and also in some reports to lysosomal function itself [97, 98, 124]. Importantly, this includes interactions with its upstream activator and fellow PD risk gene product, Rab29 [124]. The implications for endocytosis are important here, since this process is linked to polygenic risk of PD [162], as well as other PD-related genes such as *DNAJC6* and *SYNJ1* [155–157]. Nonetheless, impaired lysosomal function would also lead to an inevitable and potentially rapid increase in faulty mitochondria, as these organelles are cleared by mitophagy as part of their normal homeostasis [209]. Pathogenic *LRRK2* mutations impairing normal lysosomal function could therefore not only account for elevated levels of α-synuclein and hence Lewy bodies, but also the mitochondrial dysfunction and increased levels of reactive oxygen species that are frequently observed in neurodegeneration.

If *LRRK2* mutations cause cell death via lysosomal dysfunction, why would they affect the dopaminergic neurons of the *substantia nigra* in particular? There are many theories as to why this group of neurons are specifically lost in PD, but a curious feature of dopaminergic neurons of the *substantia nigra* is the length of their axons, which are amongst the longest of any CNS neuron. By virtue of their length, these neurons are especially sensitive to disruptions in the trafficking of organelles and vesicles over long distances. With this in mind, it is important to stress the importance of microtubules for vesicular and organellar transport. The positioning of membranous organelles is largely dictated by the cytoskeleton in all cell types, and vesicle transport along microtubules is a similarly ubiquitous phenomenon. But in neurons, axonal microtubules behave like veritable motorways, allowing communication and transport of materials between the cell body and distant growth cones [200]. We are clearly at the point of speculation, but if the key effect of *LRRK2* mutations were to be impairments in microtubule-based endolysosomal trafficking in axons, this could in a single mechanism account for lysosomal dysfunction and the ensuing effects on α-synuclein levels and mitochondria, *and* the selective involvement of dopaminergic neurons. Moreover, such a mechanism would also begin to reconcile the implication in PD from GWAS of the axonal microtubule interacting protein Tau [7], and might go some way to explaining the various signal transduction abnormalities associated with LRRK2. In the majority of cases, activated membrane receptors are trafficked through the endosomal system to lysosomes, where their signals are terminated. Manipulations that slow retrograde endosomal flux thereby increase signal duration in most pathways, although curiously, in the case of canonical Wnt signalling, where sequestration of GSK3 into late endolysosomal compartments is a key step in the activation of β-catenin, delayed trafficking of receptor signalosomes decreases signalling [210]. As mentioned, dopaminergic neurons of the ventral midbrain have a special requirement for canonical Wnt signalling in their development, and numerous lines of evidence suggest this requirement continues into adulthood [211, 212].

Our proposed mechanism is clearly not without limitations and we do not claim to have all the answers. For example, a primary deficit affecting lysosomes or late endolysosomal trafficking is not immediately reconcilable with the strong story building around LRRK2, Rab29 and the trans-Golgi network. Nonetheless, there is strong evidence that enhancing lysosomal function is strongly neuroprotective in animal PD models (e.g. [213, 214]). As such, targeting these processes seems a viable approach for reducing neurodegeneration in general, even if it does not turn out to be the best approach for targeting LRRK2 PD in particular.

## Conclusions

In many regards the LRRK2 field is the same as it has always been: complex. This protein remains at the centre of more pathways, processes and diseases than seems possible. Although breakthroughs are made, they just seem to take us back to the same place, generating even more questions. For example, in 2013 we observed that the most published facet of LRRK2 biology was interactions with microtubules [215] and the long sought after identification of Rab proteins as *bona fide* LRRK2 substrates in 2016 [34] has pointed us to two microtubular structures: centrosomes and cilia. *Plus ça change, plus c’est la même chose.* The LRRK2 field does not seem to evolve, it just seems to get bigger.

And yet the field is moving forward. Advances in structural biochemistry are taking us closer to an understanding of the structure of LRRK2 and its GTPase activity. The similarities between some of the inflammatory diseases in which LRRK2 has been implicated will likely foster advances in understanding the function of LRRK2 in immune responses – although whether PD is an inflammatory condition remains another story. And the identification of Rab29 as an activator of LRRK2 kinase will surely be a great boon to the LRRK2 community, expediting research into the pathological consequences of LRRK2 mutations, and perhaps leading to the identification of further LRRK2 substrates. Whether LRRK2 pathology turns out to be through altered cell signalling, perturbed organellar processes or impaired microtubule function – or indeed, a combination thereof – we will get there.

We also note the recent report that vitamin B_12_ is a ‘mixed-type allosteric inhibitor’ of LRRK2 kinase activity; that is, vitamin B_12_ does not compete with ATP for the active site of the LRRK2 kinase domain, as is the case for all other LRRK2 kinase inhibitors, but binds elsewhere to induce a conformational change [216]. Fascinatingly, vitamin B_12_ appears to promote the monomerisation of LRRK2, opening the door to an alternative therapeutic approach. This is potentially important, since there is evidence that conventional LRRK2 kinase inhibitors reverse the effects of pathogenic RocCOR mutations on substrate phosphorylation, but potentially *mimic* these mutations in other regards (e.g. increased LRRK2-GTP binding [64, 217], increased association with filamentous microtubular structures [217], and impaired Wnt signalling [102, 105]). The results of on-going clinical trials are therefore eagerly awaited. But in any case, when allied with conventional kinase inhibitors and guanine nucleotide binding inhibitors, vitamin B_12_ forms part of a tool kit to modulate LRRK2 function pharmacologically in three distinct ways. It is another small step towards understanding this most mysterious of proteins. These are exciting times.

## Data Availability

Not applicable

## Abbreviations

4E-BP: Eukaryotic initiation factor 4E binding protein
ANK: Ankyrin repeats
ARM: Armadillo repeats
BAG5: BCL2-associated athanogene 5
cAMP: Cyclic AMP
CD: Crohn’s disease
CK1α: Casein kinase 1α
CMA: Chaperone mediated autophagy
COR: C-terminal of roc
DVL: Dishevelled
GAD: GTPase Activated by Dimerisation
GAK: Cyclin-G–associated kinase
GAP: Guanine activating proteins
GWAS: Genome-wide association studies
Hsc70: Heatshock cognate 70
LRR: Leucine rich repeats
NFAT: Nuclear factor of activated T-cells
PD: Parkinson’s disease
PKA: Protein kinase A
RILPL: Rab interacting lysosomal protein like
Roc: Ras of complex proteins
Shh: Sonic Hedgehog
TB: Tuberculosis
TBK1: TANK-binding kinase 1
TGN: Trans-Golgi network
TLR: Toll-like receptor
GEF: Guanine nucleotide exchange factor
